# Hematoma of the psoas muscle, in prostatic cancer patient: a case report

**DOI:** 10.11604/pamj.2015.20.138.5152

**Published:** 2015-02-17

**Authors:** Ziyad Almushayti

**Affiliations:** 1 Medical Imaging Department, College of Medicine, Qassim University, Buraydah, Saudi Arabia

**Keywords:** Hematoma, prostatic cancer, ascites

## Abstract

We report a case of 64-year-old male with prostate cancer and coagulation disorder presented with abdominal distension. He underwent abdomen and pelvis ultrasound for assessment of ascites, which showed localized fluid collections identified at the left and right iliac fossa. After that, non enhanced abdomen and pelvis CT scan was performed and showed heterogeneous organized collections identified along the psoas muscles bilaterally, causing focal contour bulge representing haematoma.

## Introduction

Prostate cancer is the second most common urological malignancy to be associated with paraneoplastic syndromes after renal cell carcinoma and one of these syndromes is a hemorrhagic disorder. We report a case of 64-year-old male with prostate cancer and coagulation disorder in form of bilateral psoas muscles hematoma.

## Patient and observation

**Figure 1:**
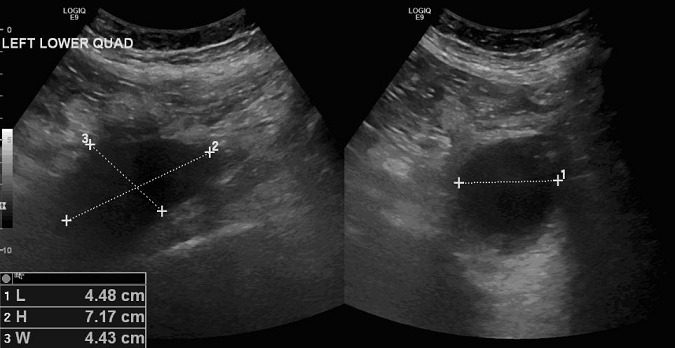
US examination showed a localized fluid collection indentified at left iliac fossa with volume approximately 74 ml

**Figure 2:**
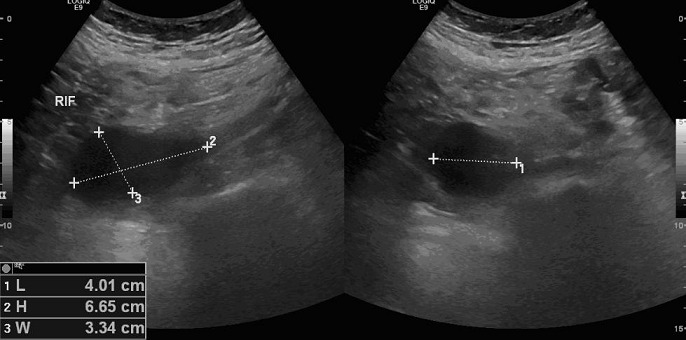
US examination showed a localized fluid collection indentified at right iliac fossa with volume approximately 48 ml

**Figure 3:**
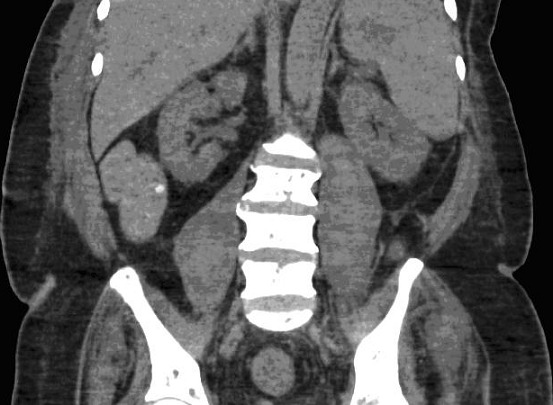
Nonenhanced CT scan examination showed heterogeneous organized collections identified along the right and left psoas muscles causing focal contour bulge representing hematoma

**Figure 4:**
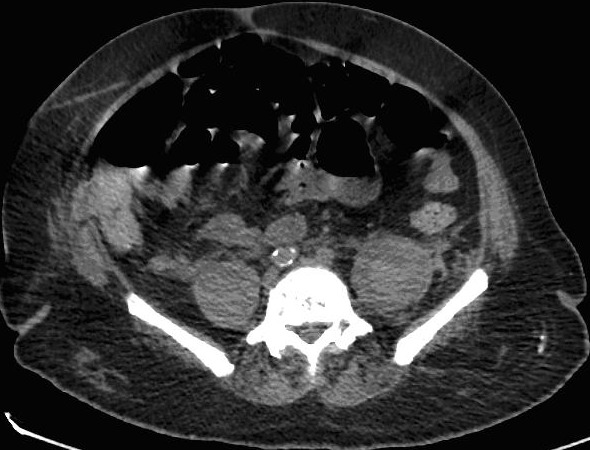
Nonenhanced CT scan examination showed heterogeneous organized collections identified along the right and left psoas muscles causing focal contour bulge representing haematoma, minimal fat stranding surrounding the psoas muscles, more significant on the left side

A 64-year-old man with prostate cancer, bone metastasis and coagulation disorder presented with abdominal distension. He underwent abdomen and pelvis ultrasound for assessment of ascitis, which showed localized fluid collections identified at the left iliac fossa with volume approximately 74 ml and right iliac fossa with volume approximately 48 ml (Figure 1,Figure 2). After that, non enhanced abdomen and pelvis CT scan was performed and showed heterogeneous organized collections identified along the right and left psoas muscles causing focal contour bulge representing hematoma, the volume is reaching approximately up to 100 ml on the right side and 150 ml on the left side. There is minimal fat stranding surrounding the psoas muscles, more significant on the left side (Figure 3, Figure 4).

## Discussion

Prostate cancer is the second most common urological malignancy to be associated with paraneoplastic syndromes after renal cell carcinoma and one of these syndromes is a hemorrhagic disorder. These syndromes tend to occur in the setting of late stage and aggressive tumors with poor overall outcomes. Paraneoplastic syndromes represent a constellation of conditions that are caused by the presence of malignancy, but not attributable to direct tumor invasion or compression. Up to 8% of patients with cancer are estimated to be affected by paraneoplastic syndromes [1]. Recognition of these syndromes is clinically important as it might lead to the detection of underlying malignancy and impact on the treatment options available. Over 70% of cases document the syndrome as the initial clinical manifestation of prostate cancer, while in just fewer than 20% the syndrome was an initial sign of disease progression to the castrate-resistant state. The causes of paraneoplastic syndromes in prostate cancer are incompletely understood [1]. The spectrum of hematological disorders associated with prostatic malignancy ranges from acute bleeding diathesis to thrombosis and embolic events. Disseminated intravascular coagulation (DIC), however, is the disorder most commonly observed in patients with prostate cancer. DIC can be chronic or acute in nature and is characterized by the increased production of fibrin, increased fibrinolysis and unchecked coagulation throughout the systemic circulation [1]. Thrombin activation can cause microthrombosis at the small-vessel to mid-vessel level [1]. Over time, the clotting proteins in blood are “used up.” When this happens, a higher risk for serious bleeding. Also, consumption of coagulation factors and aggressive fibrinolysis can cause acute bleeding. It has been suggested that subclinical DIC is relatively common in patients with metastatic prostate cance [1]. So, hematoma of the psoas was caused by disseminated intravascular coagulation arising from multiple bony metastases of a prostatic cancer [2]. Clotting profiles should be monitored closely in patients with prostate cancer and paraneoplastic DIC, as abnormalities might need to be corrected before or after biopsy [1]. Treatment of hematological paraneoplastic disorders is two-fold: management of the underlying prostate cancer and supportive therapy for the coagulation disorder [1].

## Conclusion

Hematoma of the psoas muscle in prostate cancer patient is generally secondary to a coagulation disorder. Treatment is two-fold: management of the underlying prostate cancer and supportive therapy for the coagulation disorder.
